# Revised diagnostic criteria for Vascular Cognitive Disorders: the VASCOG‐2 criteria

**DOI:** 10.1002/alz.087175

**Published:** 2025-01-03

**Authors:** Perminder S. Sachdev, Adam C. Bentvelzen, VasCog‐2 Criteria Consortium

**Affiliations:** ^1^ Neuropsychiatric Institute, Prince of Wales Hospital, Randwick, NSW Australia; ^2^ Centre for Healthy Brain Ageing (CHeBA), University of New South Wales, UNSW Sydney, NSW Australia; ^3^ Centre for Healthy Brain Ageing (CHeBA), UNSW Sydney, Sydney, NSW Australia

## Abstract

**Background:**

Several sets of diagnostic criteria have been published for vascular cognitive disorders (VCD) since the 1960s. The International Society for Vascular Behavioral and Cognitive Disorders (VASCOG) working group published a comprehensive and operationalized criteria set in 2014. Considering the significant developments in the field in the last decade, an international expert group was commissioned to revise these criteria using the Delphi process.

**Methods:**

A modified Delphi consensus method was used, involving an iterative, multi‐staged series of structured surveys with feedback of anonymized responses from experts in the fields of cerebrovascular disease and dementia. Three rounds were planned, with the possibility of a fourth round, if required to reach consensus. Literature reviews on topics related to the criteria were conducted by a team of researchers, which informed the first structured questionnaire. Each salient point in the criteria was put to consensus, and relevant recent reviews and publications provided for background information. Consensus was defined as agreement or disagreement on any statement of ≥ 75%, near consensus as 66 – 75%, and <66% as non‐consensus. Statements that reached consensus were removed from subsequent rounds, as were most that had non‐consensus, with some being reworded. One in‐person meeting in Gothenburg, Sweden and one virtual meeting were held for experts to discuss contentious issues and advise on the process.

**Results:**

Seventy experts from a broad range of international regions and disciplines consented to participate, and 54 completed the Round 1 survey. The final round is being completed, after which the revised criteria will be finalized for formal approval by all participants. In addition to the criteria for clinical use, which include neuroimaging criteria, a set for research use will be agreed upon.

**Conclusions:**

The updated VASCOG‐2 criteria could serve as the international standard for VCD diagnosis, thereby facilitating diagnostic consistency between clinicians and researchers.

